# Coaxial 3D bioprinting of self-assembled multicellular heterogeneous tumor fibers

**DOI:** 10.1038/s41598-017-01581-y

**Published:** 2017-05-03

**Authors:** Xingliang Dai, Libiao Liu, Jia Ouyang, Xinda Li, Xinzhi Zhang, Qing Lan, Tao Xu

**Affiliations:** 1 Department of Neurosurgery, the Second Affiliated Hospital of Soochow University, Suzhou, 215004 China; 2 Department of Mechanical Engineering, Biomanufacturing Center, Tsinghua University, Beijing, 100084 China; 3 Medprin Biotech GmbH, Gutleutstraße 163-167, Frankfurt am Main, 60327 Germany; 4 Department of Precision Medicine and Healthcare, Tsinghua-Berkeley Shenzhen Institute, Shenzhen, 518055 China

## Abstract

Three-dimensional (3D) bioprinting of living structures with cell-laden biomaterials has been achieved *in vitro*, however, some cell-cell interactions are limited by the existing hydrogel. To better mimic tumor microenvironment, self-assembled multicellular heterogeneous brain tumor fibers have been fabricated by a custom-made coaxial extrusion 3D bioprinting system, with high viability, proliferative activity and efficient tumor-stromal interactions. Therein, in order to further verify the sufficient interactions between tumor cells and stroma MSCs, CRE-LOXP switch gene system which contained GSCs transfected with “LOXP-STOP-LOXP-RFP” genes and MSCs transfected with “CRE recombinase” gene was used. Results showed that tumor-stroma cells interacted with each other and fused, the transcription of RFP was higher than that of 2D culture model and control group with cells mixed directly into alginate, respectively. RFP expression was observed only in the cell fibers but not in the control group under confocal microscope. In conclusion, coaxial 3D bioprinted multicellular self-assembled heterogeneous tumor tissue-like fibers provided preferable 3D models for studying tumor microenvironment *in vitro*, especially for tumor-stromal interactions.

## Introduction

3D bioprinting has reflected its significant advantages in the fabrication of tissue engineered constructs with its characteristics of excellent scalability, repeatability and precise deposition positioning, which are unattainable with traditional fabrication techniques^1^. Various printing technologies, such as inkjet-based bioprinting, laser-assisted bioprinting, and pressure-assisted bioprinting have been employed to fabricate 3D living constructs. However, each method has its own advantages and limitations^2^. For inkjet-based bioprinting, droplets of cell-laden bioink are generated at the narrow nozzle by a thermal or piezoelectric actuator, the resulting shear stress would negatively affect cell viability due to damage to the cell membrane and cell lysis. Laser-assisted bioprinting is a method that uses laser as the energy source to deposit cell encapsulating hydrogel droplets, but parameters such as ink bubble dynamics, laser pulse energy and shear stress all influent the bioprinting process. In pressure-assisted bioprinting, cell-laden biomaterials are extruded by pneumatic pressure or plunger- or screw-based pressure through a micro-scale nozzle to fabricate 3D constructs layer by layer. It enables printing of a wide array of bioinks, not only cell-laden hydrogels, but also cell aggregates, micro-carriers and decellularized matrix components. However, the resolution of this method is generally greater than 100 μm, and the shear stress from the nozzle tip wall significantly affects cell viability. Regardless of which bioprinting technique is used, the high viability and intrinsic function of the cells mixed in the hydrogel are always the goals being pursued.

In addition to bioprinting methods, hydrogel properties are relevant to bioprinting applications, such as gelation time, swelling or contraction, stability, biocompatibility and printability^3^. The physical properties (rigidity, strength, viscosity, and adhesion), chemical properties (degradation rate, degradation product toxicity), and immunogenicity of the bioink materials have significant impacts on cell biological functions such as motility, migration, differentiation and even gene expression^4^. Some impacts are favorable factors for cell adhesion, cell intrinsic functions and their applications, but some are not. The most commonly used material, sodium alginate sol solution, has the desired viscosity and printability. However, the rigidity and stiffness of it increase when crosslinked by CaCl_2_ and hydrogel forms. Although it may be better than the 2D model, cells mixed in are subject to considerable restrictions such as lack of mobility and cell-cell interactions. For 3D spatial living tissues bioprinted *in vitro*, sufficient cell-cell connections and interactions are the most necessary conditions for a biomimetic microenvironment^5, 6^. Currently, it is still challenging to achieve desired cell-cell interactions for alginate-based bioprinting, which restricts its development in tissue engineering and tumor research.

As for tumor models, traditional 2D culture models and animal models are limited in many aspects^7^. Various 3D tumor models have been created and applied. 3D cultured multicellular layer model mimics the tumor heterogeneity to some extent but still presents as a planar geometry^8^. Gel or matrix embedding 3D tumor models are advantageous in maintaining the 3D architecture but limited in mimicking the mass transport gradient of the tumor environment^9^. Hollow fiber bioreactor systems have the ability to generate solid masses in capillaries used for metabolism and cancer cell resistance, but the fiber wall limits the growth of tumors^10^. Multicellular spheroids are most widely used tumor models *in vitro*, because they integrate various features of the *in vivo* tumor microenvironment. However, some limitations also exist. For example, the dissociation of tumor spheroids, and it is hard for the exchange of cellular metabolites and maintaining of accurate repeatability of the cells in the spheroids^11^. With the development of 3D bioprinting technology, 3D bioprinted *in vitro* tumor models have also been developed and applied^12^. Xu *et al*. reported the first bioprinted tumor model of human ovarian cancer (OVCAR-5) cells together with normal fibroblasts, providing a promising method for the research of tumor-stroma interactions^13^. Burks and colleagues used laser direct-write technique to create a model by bioprinting cancer cell and/or stromal fibroblasts onto rat mesentery tissue for cancer cell migration and invasion research^14^. Also, Leonard *et al*. reported the development of bioprinted *in vitro* breast cancer co-cultured model^15^. Zhao *et al*. bioprinted Hela cells with gelatin/alginate/fibrinogen hydrogel and found that MMP-2 and MMP-9 expression of tumor cells in hydrogel were higher than those of the 2D control^16^. Previously, we have bioprinted a brain tumor model by extruding glioma stem cells-laden hydrogel^17^. But it is known that solid tumor contains not only tumor cells but also a variety of stromal cells, which are the important sources of extracellular matrix and cytokines. For example, in glioma microenvironment, glioma stem cells (GSCs) and mesenchymal stem cells (MSCs) are the most important two groups of cells^18^. The former is considered to be the source of glioma occurrence and recurrence^19^, the latter is considered to promote tumor progression^20^. Moreover, MSCs and their differentiated fibroblasts can secrete extracellular matrix (ECM) and participate in the process of tumor remodeling, which plays an important role in the biologic fabrication of tumor tissue *in vitro* ^21^. Multicellular heterogeneous tumor model with sufficient tumor-stroma interactions are ideal for this purpose.

In this study, a multicellular heterogeneous tumor model of cell-laden “core-shell” construction was fabricated by the coaxial 3D bioprinting system, using alginate/gelatin (A/G) as the external shell, and cell suspension containing fibrinogen as the core (CoF). In the core, red fluorescence protein (RFP)-expressing glioma stem cells (GSCs) and green fluorescence protein (GFP)-expressing mesenchymal stem cells (MSCs) were used for observing tumor-stroma interactions and tumor fibers self-assemble. Furthermore, tumor-stroma cell fusion was demonstrated by using the “CRE-LOXP system”^22^, in which GSCs were transfected with “LOXP-STOP-LOXP-RFP” gene and MSCs contained CRE gene. When tumor-stroma cell fusion occurs, CRE enzyme will cut off the two LOXP sites as well the STOP site contained in between, and then the RFP gene can be expressed. Two-dimensional group (2D group) and the group of cells mixed directly into hydrogel (Mixed group) were used as controls, to compare the interaction between GSCs and MSCs under different conditions.

## Results

### Coaxial bioprinting system and characteristics the of A/G shell

**Figure 1:**
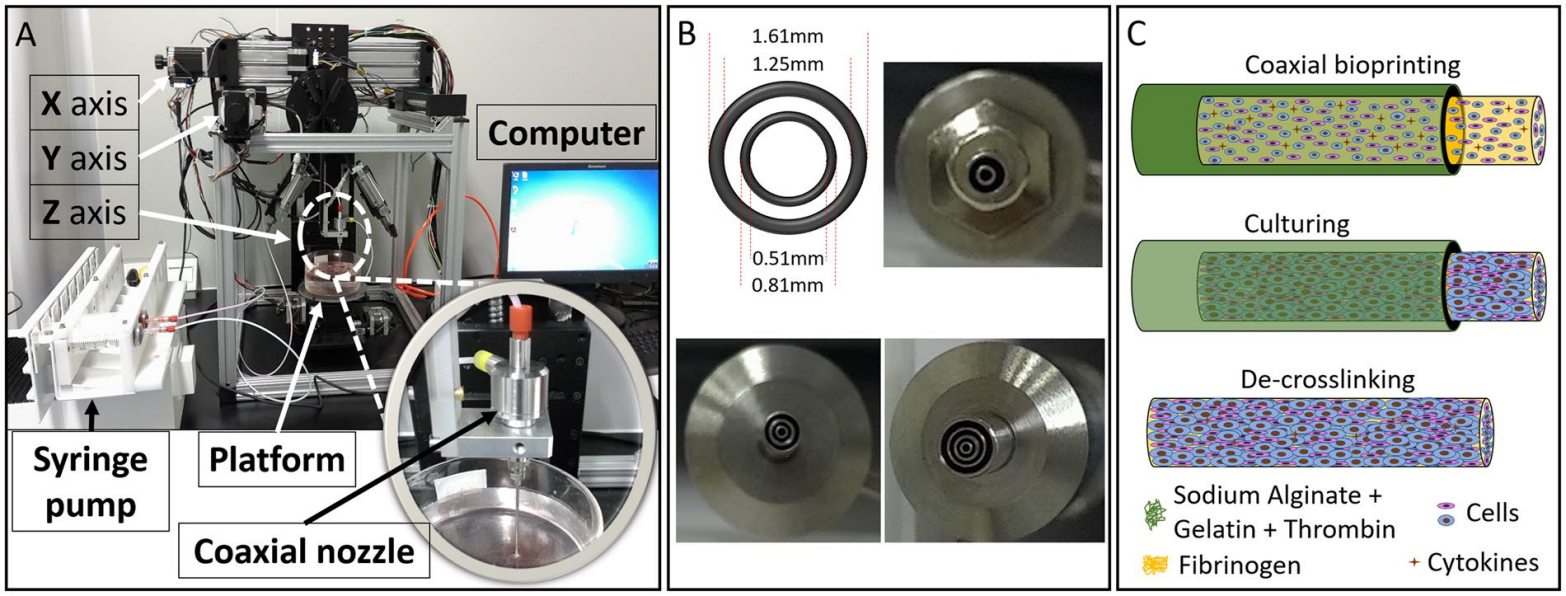
Coaxial 3D bioprinting system and the tumor fiber fabrication process. (**A**) Coaxial 3D bioprinting system contains a XYZ three-axis platform, a syringe pump, a coaxial nozzle and the computer-controlled system. (**B**) Different styles of the printing nozzles adapted with this bioprinting platform. (**C**) The schematic of the biofabrication process of multicellular heterogeneous tumor fiber: coaxial bioprinting, *in vitro* culturing and de-crosslinking.

Core-shell structures were bioprinted through a custom-made computer-controlled coaxial extruding bioprinting system, including a three-axis motion platform, a syringe pump, a coaxial nozzle and a computer-controlled system (Fig. 1A). Coaxial nozzle composed of a 21G inner nozzle (0.81 mm and 0.51 mm for outer and inner diameter, respectively) and a 16G outer nozzle (1.61 mm and 1.25 mm for outer and inner diameter, respectively). This can be expanded to tri-axial or multi-axis nozzles with different inner and outer diameters to facilitate the manufacture of more complex structures (Fig. 1B). Scaffold-free tissue fibers can be achieved after washing out the shell with sodium citrate and ethylene diamin tetra-acetate (EDTA) solution. Schematic diagram of this process is shown on Fig. 1C.

**Figure 2:**
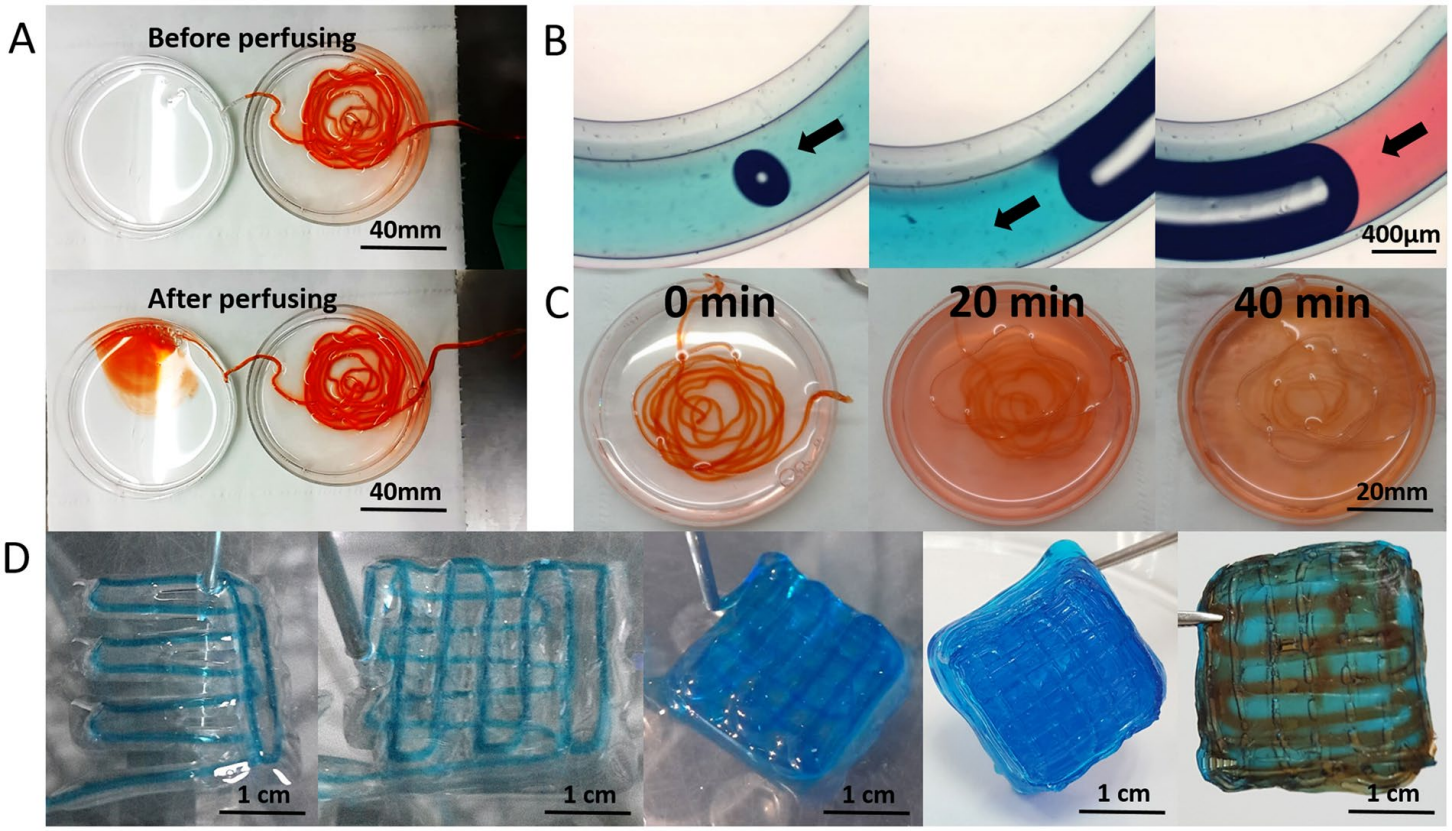
Characteristics of the core-shell structures. (**A** and **B**) Integrity and continuity of the extruded liquid flow: the solution in the core stained with red or blue dyes can flow through the printed fiber shell without leaking out; (**C**) Permeability: Dye can uniformly diffuse out the structure within 40 minutes; D Layer by layer bioprinting (left 1–4) and perfusion of the printed construct with red dyes (right 1). Scale bars: (**A**) 40 mm, (**B**) 400 μm, (**C**) 20 mm, (**D**) 1 cm.

To evaluate the feasibility of this experiment, the integrity and permeability of the A/G shell was tested. Core was replaced with 3% CaCl_2_ solution, added with red (FD&C red NO. 1) and blue (FD&C BLUE NO.1, Vidhi Dyestuff Manufacturing Limited, Tardeo, Mumbai, India) dyes in the liquid flow in core. Results showed that the coaxial bioprinting system can extrude shell structures, and their integrity can be maintained even at high liquid flow rate by perfusing manually (Fig. 2 Aup before perfusing, Fig. 2 Adown after perfusing, and Fig. 2B). To evaluate the permeability, shell structures filled with red liquid dye in core were incubated in 0.9% NaCl solution. Results showed that the A/G shell structure has excellent penetrability, as the red dye diffused uniformly after 40 minutes of soaking (Fig. 2C). This indicates that small molecule nutrients, cytokines and oxygen can transport freely across the shell. Higher concentrations of the alginate solution could result in stronger shell structure with high rigidity, which is beneficial for supporting the core. However, when the alginate concentration is too high, the resulting hydrogel could have high crosslinking density that would hinder cell motility and diffusion of macromolecules. Ma *et al*. has reported that the model biomacromolecule EGFP can diffuse freely in 4% alginate microchannels^23^, thus, it is concluded that macromolecules can also transport freely across the shell reported here, which was made from 3% alginate solution. In addition, results showed that meter-long fibers can not only be extruded from the bioprinting platform, but also be stacked layer by layer. After printing, the core can be perfused with cell suspension, as well as with medium (Fig. 2D).

### 3D bioprinted core-shell cell fibers

**Figure 3:**
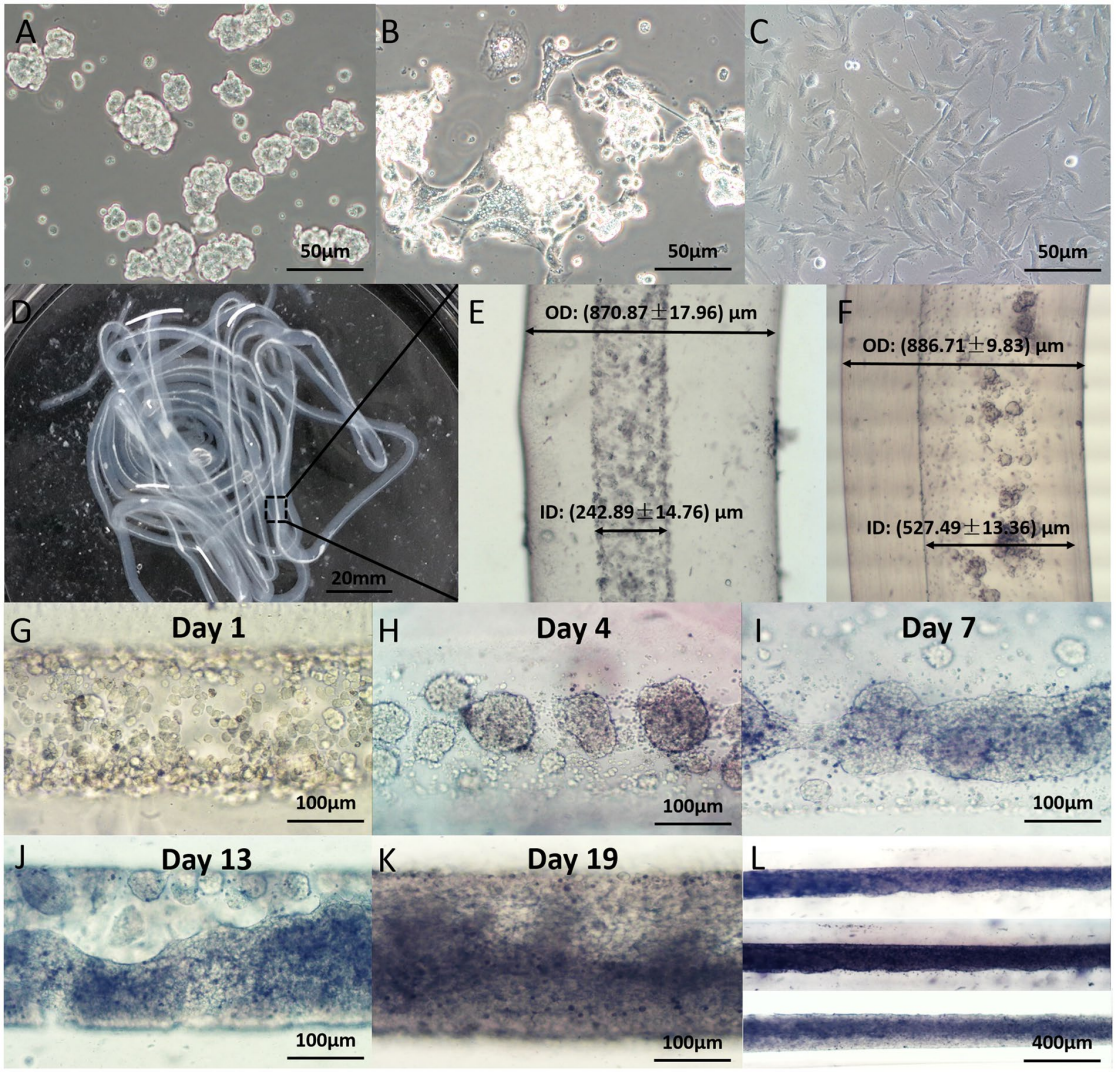
3D core-shell cell fibers. (**A** and **B**) GSC23 grew in stem cell medium (**A**) and complete medium (**B**); C: MSCs cultured in complete medium; D: Coaxial 3D bioprinted cell fibers; (**E** and **F**) Cell fiber with extrusion speed of 3 ml/h and 10 ml/h; (**G**–**L**) Coaxial 3D bioprinted tumor fiber cultured for 1, 4, 7, 13, and 19 days. Scale bars: (**A**–**C**) 50 μm, (**D**) 20 mm, (**G**–**K**) 100 μm and (**L**) 400 μm.

Glioma stem cells, GSC23 grew in spheres in stem cell medium (Fig. 3A), but grew with adherence to the 2D substrate when fetal bovine serum (FBS) was added (Fig. 3B). With A/G as the shell, high density cell suspension as the core, 3D core-shell cell fiber structures were bioprinted (Fig. 3D). It is very important to have proper extrusion parameters for the core suspension: when the extrusion rates of cell suspension and A/G were 3 ml/h and 30 ml/h, respectively, the inner and outer diameters of the A/G shell were (242.89 ± 14.76) μm and (870.87 ± 17.96) μm, respectively (Fig. 3E); when the extrusion rate of cell suspension increased to 10 ml/h, the inner and outer diameters of the shell were (527.49 ± 13.36) μm and (886.71 ± 9.83) μm, respectively (Fig. 3F). Thus, by adjusting the extrusion speed, inner diameter of the shell, or the diameter of core cell fiber, could be changed while the outer diameter remained unchanged. This phenomenon was in exact agreement with what has been reported previously^24^.

The shell thickness is an important parameter for the core-shell structure. On one hand, an ideal structure should have thin shells for increased permeability and easy delivery of nutrients. On the other hand, thick shells are needed for structural integrity and to provide a concentration gradient that properly mimics the *in vivo* tissue environment. The growth of the tumor and stromal cells in the fibers also has distinct features. As shown on Fig. 3G–L, first cells gathered into spheroids, then multicellular spheroids connected to each other, and integrated into fibers. Finally the fibers fused into tissue-like structures filling up the entire core space (Fig. 3H,I).

### Cell viability and proliferation

**Figure 4:**
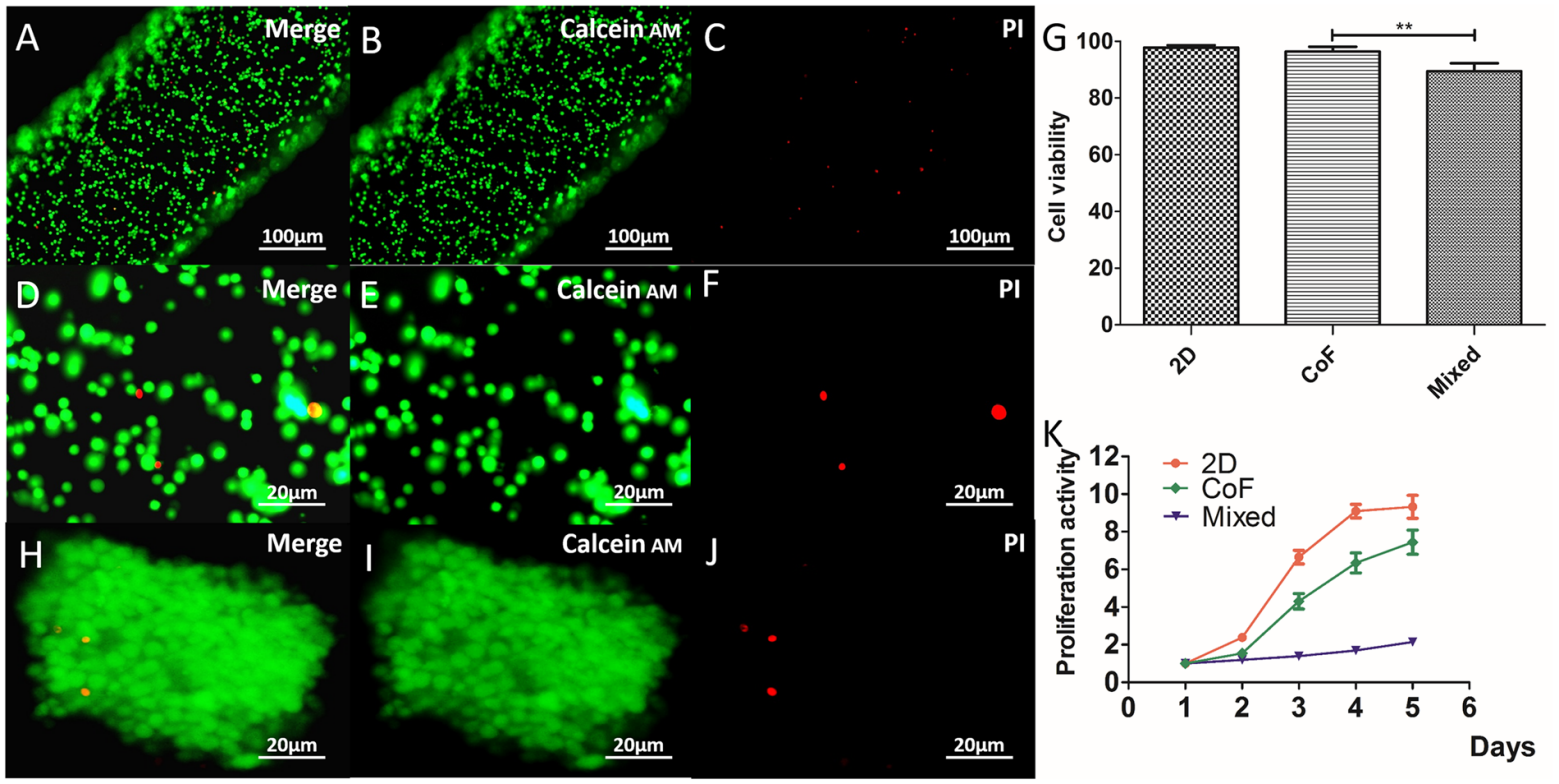
Cell viability and proliferation. (**A**–**F**) Live/dead assay for cell viability immediately after bioprinting; (**G**) Cell survival rate of CoF group comparing to cells without bioprinting; (**H**–**J**) Cell viability after culturing for 5 days; (**K**) Cell proliferation of CoF, 2D and mixed group after normalized to OD value of day 1. Scale bars: (**A**–**C**) 100 μm, (**D**–**F**) 20 μm, (**H–J**) 20 μm.

After bioprinting, live/dead assay showed that almost all of the cells in the core remained alive and stained green. Little amount of dead cells, stained positive with PI (red) were observed (Fig. 4A–F). Cell survival rate was 96.36 ± 1.54% on average, which was similar to that of cell suspension control at 97.75 ± 0.77%, but higher than that of the mixed group at 89.46 ± 2.51% (Fig. 4G). After *in vitro* culturing for 5 days, cells gathered into masses, while maintained their high viability (Fig. 4H–J). CCK-8 assay showed that the proliferation rate of the CoF group was lower than that of the 2D group, but was significantly higher than that of the mixed group (Fig. 4K).

### Self-assembled multicellular heterogeneous brain tumor fibers

**Figure 5:**
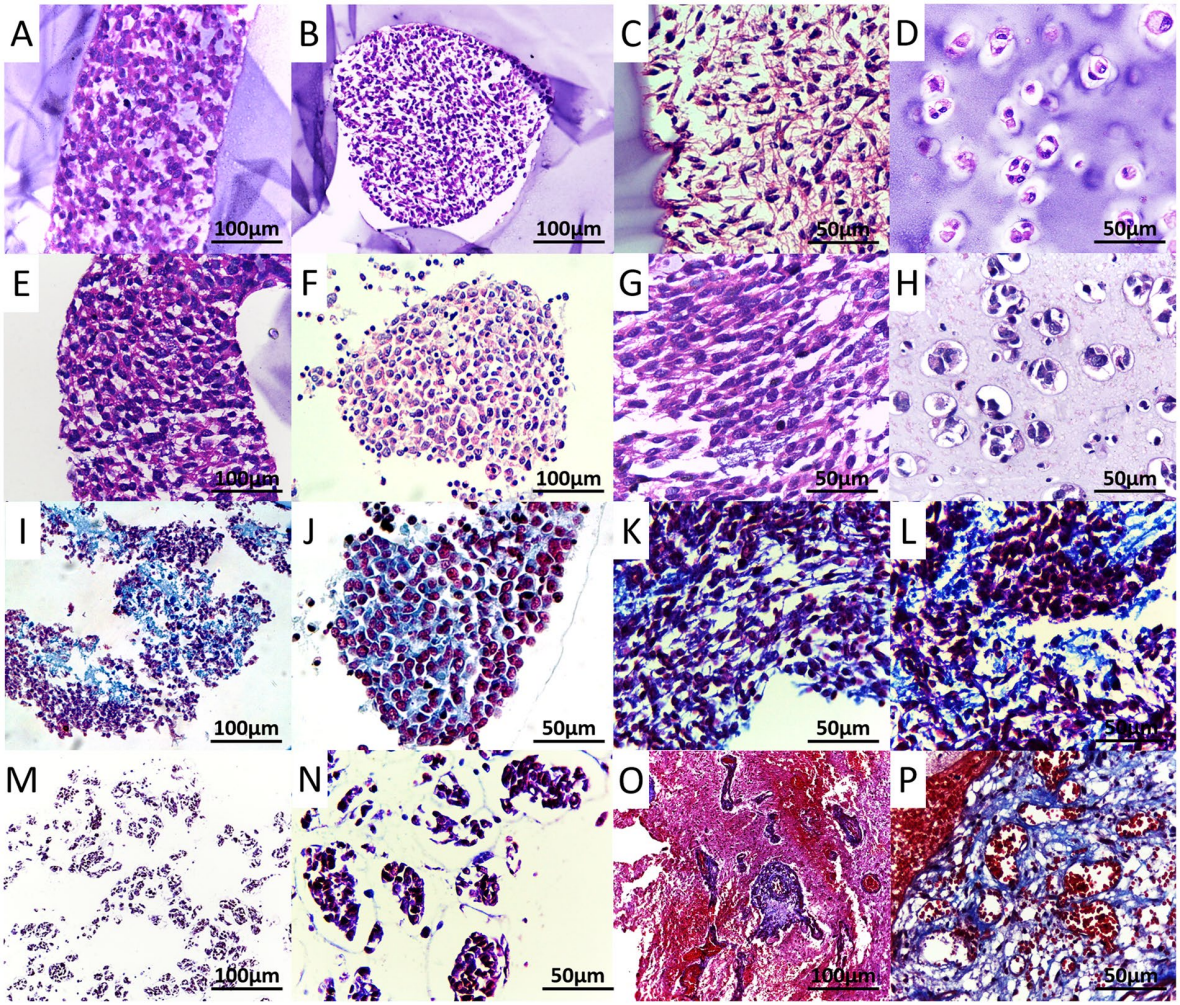
HE and Masson stainings. (**A**–**C**) The coaxial 3D bioprinted tumor fiber within the alginate shell; (**D**) Cells mixed into alginate and cultured for 7 days; E-G: HE staining of the tumor fiber after removal of the alginate shell; H: Cells mixed into alginate and cultured for 21 days. (**I**–**L**) Masson staining of the tumor fiber; (**M**,**N**) Masson staining of the cells mixed into alginate; (**O**,**P**) Masson staining of the GBM tissues. Scale bars: (**A**,**B**,**E**,**F**,**I**,**M** and **O**) 100 μm; (**C**,**D**,**G**,**H**,**J**–**L**, **N** and **P**) 50 μm.

Cell-laden core-shell structures were immersed into stem cell medium supplemented with 10% FBS, and cultured for 14 days *in vitro*. Hematoxylin-eosin (HE) staining showed that cells packed densely with cell cytoplasm being stretched (Fig. 5A–C), and the morphologies were different from that of cells cultured in alginate hydrogels (Fig. 5D,H). After removal of the alginate shell, cell fibers were still able to maintain their structures (Fig. 5E–G). Furthermore, Masson staining was performed to demonstrate the production of collagen fibers, one of the major components of extracellular matrix (ECM). Results showed that cells in the tumor fibers produced collagen fibers (stained blue) but cells mixed in the alginate did not (Fig. 5I–N). Glioblastoma (GBM) tumor tissues were used as control (Fig. 5O,P). It is indicated that ECM has been produced spontaneously and promoted the formation of self-assembled heterogeneous tissue-like brain tumor fibers.

**Figure 6:**
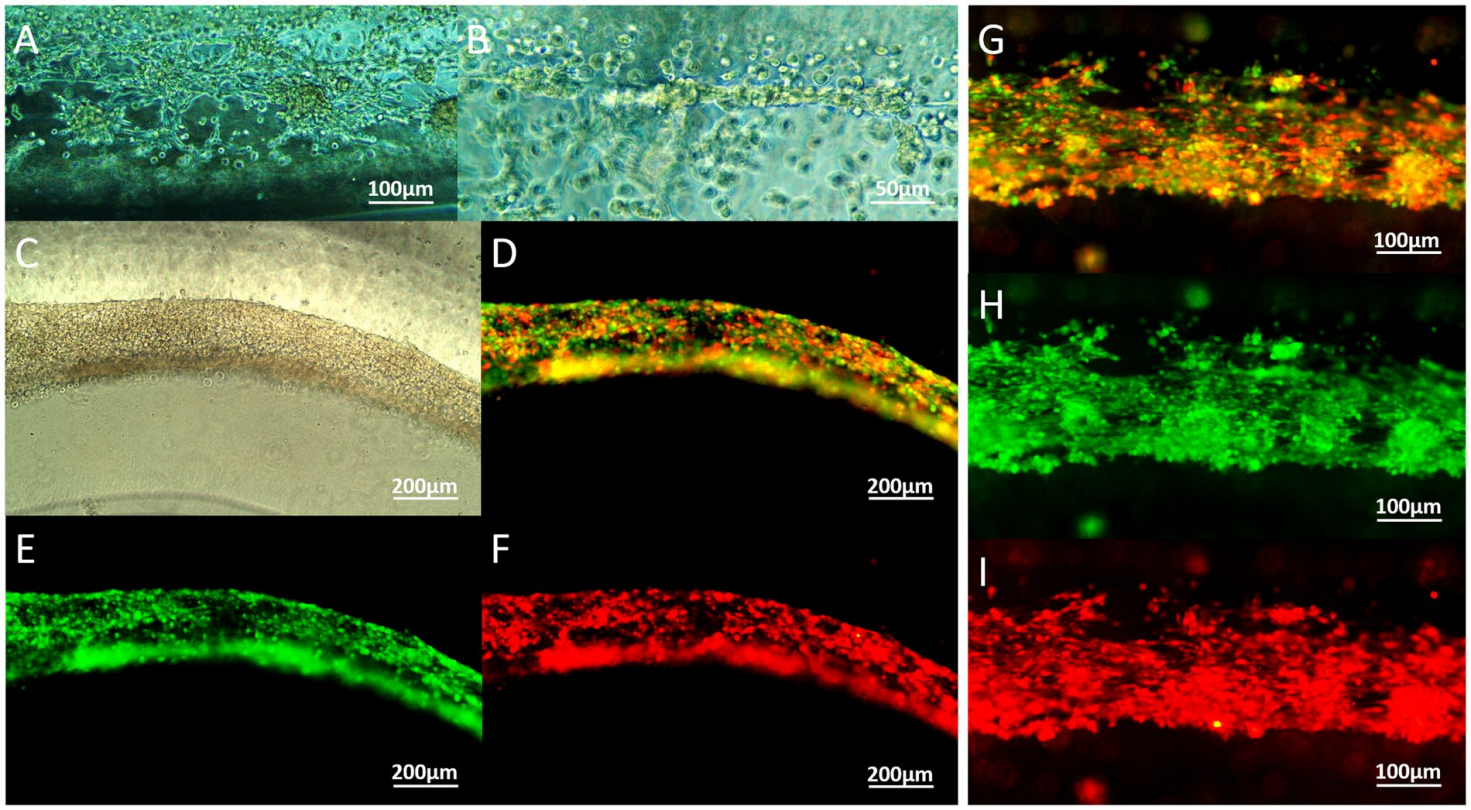
RFP/GFP traced tumor/stroma cell fibers. (**A**,**B**) Tumor cells and stroma cells fused into flakes and strands on day 2; (**C**–**F**) Cell fibers containing RFP-expressing tumor cells and GFP-expressing MSCs after bioprinting and cultured *in vitro* for 3 days; (**G**–**I**) Cell fibers cultured *in vitro* for 7 days. Scale bars: (**A** and **G**–**I**) 100 μm; (**B**) 50 μm; (**C**–**F**) 200 μm.

In order to observe self-assembly and interactions between tumor cells and stroma cells, RFP-expressing glioma stem cells and GFP-expressing MSCs were mixed into 3% fibrinogen and bioprinted in the core. On the second day, tumor cells and MSCs integrated into flakes and strands (Fig. 6A and B). Under fluorescence microscope, it was observed that RFP-expressing tumor cells and GFP-expressing MSCs were mixed and arranged in fibers (Fig. 6C–F). After 7 days, cells stretched, and integrated into fibers (Fig. 6G–I).

**Figure 7:**
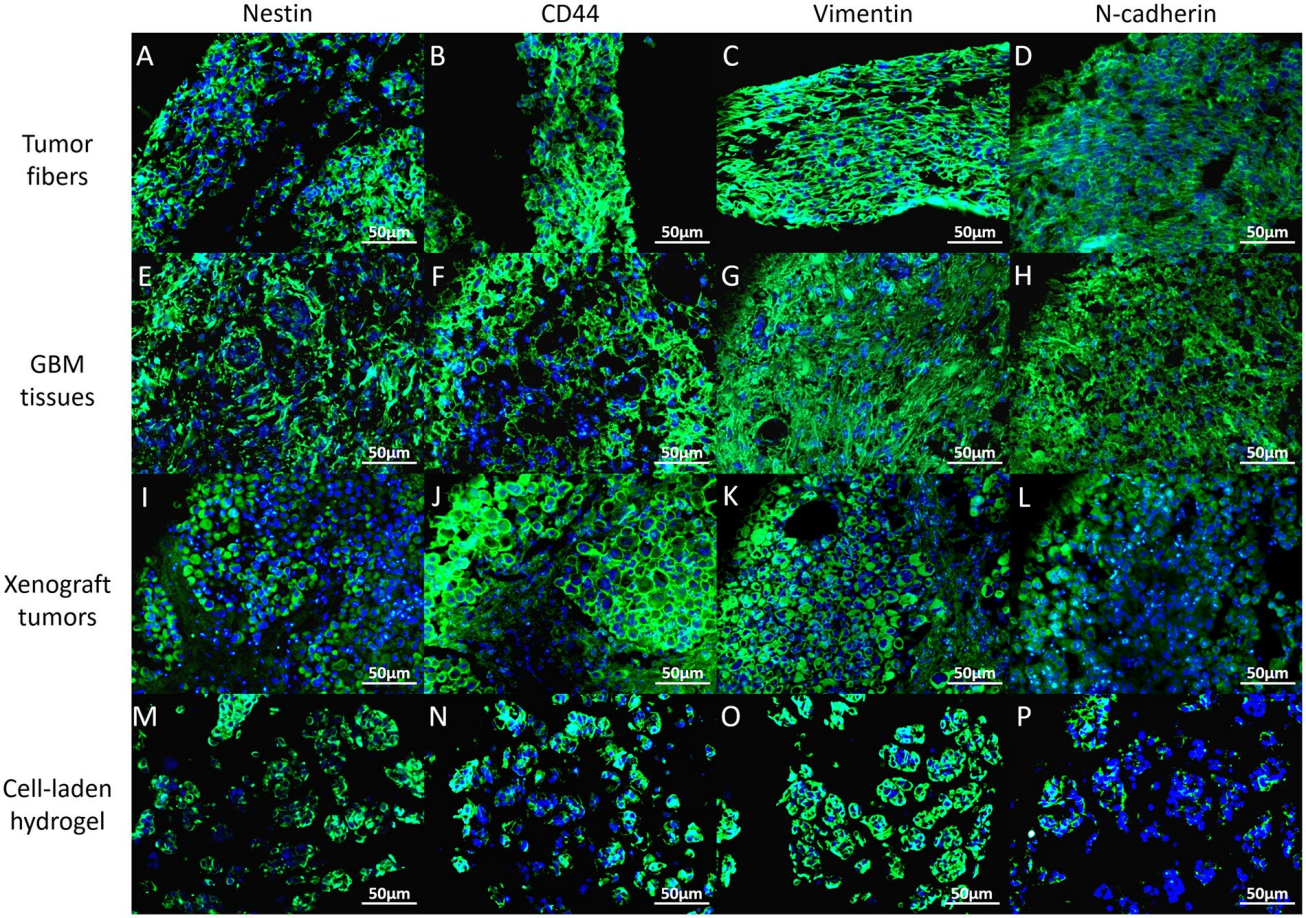
Immunohistology. Expression of biomarkers (Nestin, CD44, Vimentin and N-cadherin) in coaxial bioprinted tumor fibers, comparing to GBM tissues, xenograft tumors and cell-laden hydrogel (cells mixed into A/G). Scale bars: 50 μm.

Coaxial bioprinted tumor fibers had high expression of the glioma stem/progenitor cell biomarker Nestin (Fig. 7A), mesenchymal stem cell biomarkers CD44 and Vimentin (Fig. 7B and C) comparing to the cells mixed in alginate hydrogel. Immunofluorescence analysis also showed high expression of N-cadherin (Fig. 7D). The expression of these markers in cell fibers were comparable to that of GBM tissues and xenografted tumors (Fig. 7E–L), and was higher than that of cells mixed into alginate (Fig. 7M–P), especially the expression of N-cadherin (Fig. 7P). Cadherin mediates the interactions between tumor cells and ECM and enables an anchorage/adhesion dependent survival of cancer cells^25^. Expression of these cell markers indicated that the characteristics and functions of these cells remained unaltered, which are the basis of the self-assemble of cell fibers *in vitro*.

### Cell fusion between tumor cells and stromal cells

**Figure 8:**
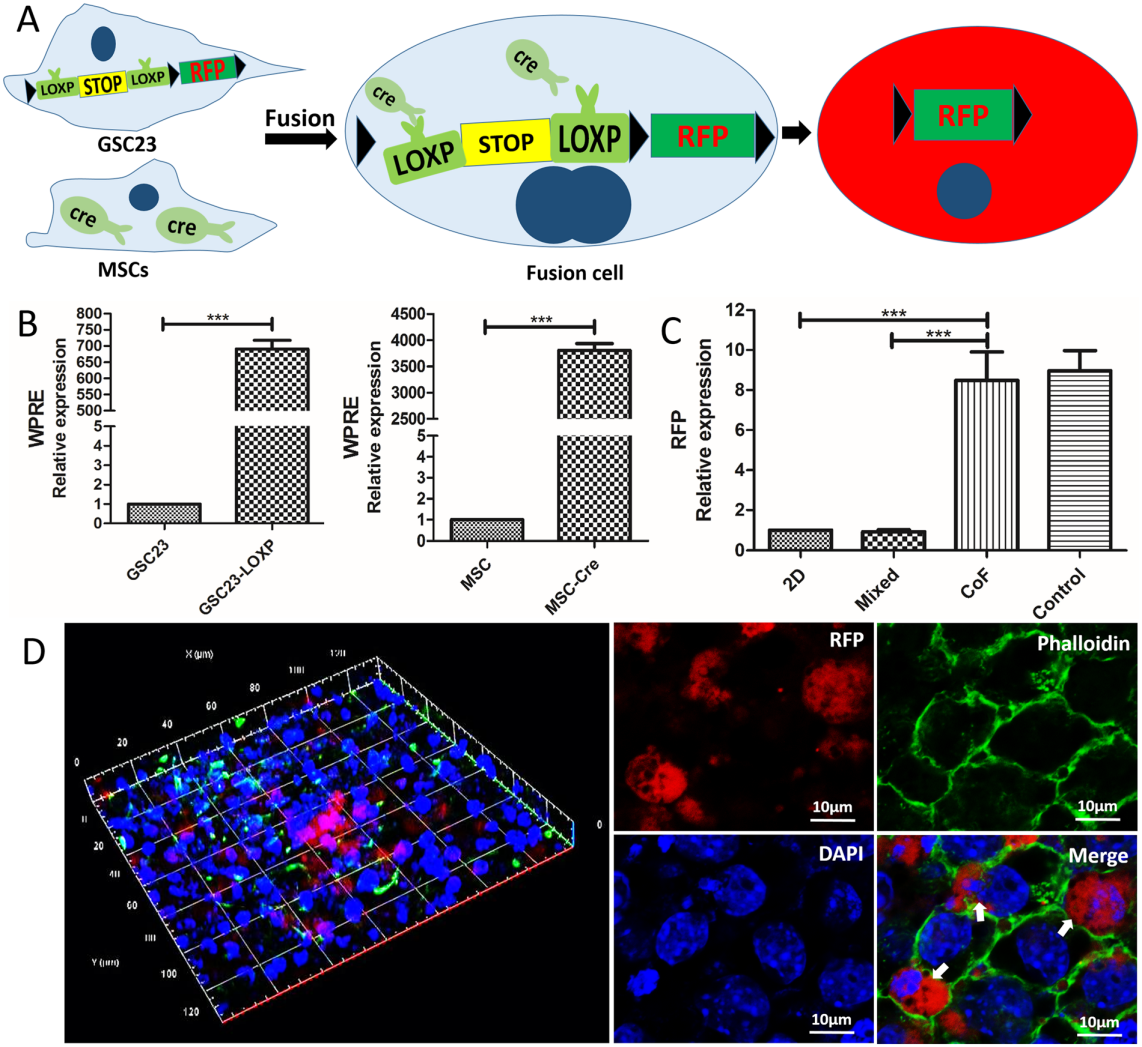
Cell fusion. (**A**) Schematic of interaction principle between CRE enzyme and LOXP-STOP- LOXP –RFP gene; (**B**) qRT-PCR validation of the transfection efficiency in GSC23 and MSC cells; (**C**) qRT-PCR validation of the RFP expression in CoF, comparing to 2D group, mixed group and coaxial group without adding fibrinogen to cell suspension (control); (**D**) Confocal images of GSC23-MSCs fibers, red is RFP (white arrow), phalloidin was used to stain the cytoskeletal structures (green) and DAPI was used to stain the cell nucleus (blue). Scale bars: (**D**) 10 μm.

CRE-LOXP system was used to compare the interactions between tumor and stroma among 2D, Mixed, CoF and Coaxial groups by detecting cell fusion. As shown in Fig. 8A, First, viral vectors were successfully transfected into cells by verifying the expression of WPRE gene, one of the gene elements contained in the viral vectors: transcription of WPRE in transfected GSC23 cells (GSC23-LOXP) was (690.92 ± 21.69) times higher than that of GSC23 not transfected; (Fig. 8B) transcription of WPRE in transfected MSCs (MSC-Cre) was (3805.58 ± 107.22) times higher than that of MSCs without transfection. After bioprinting and cultured *in vitro* for 7days, GSC23 cells and MSCs contacted and interacted with each other. Transcription and expression of RFP were evaluated by qRT-PCR and confocal microscopy, respectively. As shown on Fig. 8C, average RFP transcriptional level of CoF group was (8.48 ± 1.01) and (8.96 ± 0.71) times higher than that of 2D culture model and control group with cells mixed directly into alginate, respectively; and coaxial group (only cell suspension in core without fibrinogen) was used to justify that the addition of fibrinogen will not affect the interactions between cells. Cells mixed into the alginate had transcription level as low as that of the 2D group (0.93 ± 0.07), resulting in little communication between tumor cells and stromal cells due to the presentence of biomaterials. The presence of RFP in CoF cell fibers was observed by confocal microscopy, with phalloidin and DAPI staining the cytoskeletal and nuclei, respectively. As shown on Fig. 8D, RFP was observed in cytoplasm of the cells, verifying the communication between tumor cells and stromal MSCs, while RFP was not observed in the control group (Supplemental Fig. 1).

## Discussions

Coaxial extrusion is a promising method that could provide sufficient cell-cell interactions in 3D space^26^. With coaxial bioprinting, a “core-shell” structure can be biofabricated with the shell being the scaffold and the core being high density cell suspension. Furthermore, the material system of the shell portion can be dissolved by mild condition to obtain scaffold-free tissue-like fibers^27^. With the help of the printing platform technology, “core-shell” cell fiber structures can be fabricated layer-by-layer with predesigned shape. The shell has sufficient strength to serve as a scaffold and the core is soft which is beneficial for cell survival^28^. This superior biological structure lays the foundation for its applications, such in as tissue engineering and as drug release platforms^29^. Also, the thick and porous cell-laden structures are sufficient for supporting initial cell viability, as well as supporting functions, such as vascularization, differentiations and tissue heterogeneity^30–32^. By controlling the fiber diameter and shell thickness, the “core-shell” structure can simulate the concentration gradient of infiltrated tissue fluid, to meet the needs of nutrients and bioactive molecules supply, and will not prevent the retention of soluble metabolic waste^33^. Onoe *et al*. produced core-shell cell-laden fibers by coaxial bioprinting, and suggested that it is a preferred method to fabricate tissues that are highly mimetic of *in vivo* intrinsic morphologies and functions^26^. For tumor research, biomimetic tumor microenvironment can also be achieved by the coaxial 3D bioprinting platform, in which tumor cells and stroma cells interacted with each other and secreted ECM, growth factors, and hormones^34^. Thus, physiological and pathological interactions between tumor-stroma cells and cells-ECM can be understood more directly, paving the way for the tumor research.

In this study, a multicellular heterogeneous brain tumor fiber was fabricated by the coaxial 3D bioprinting platform (Fig. 1). First, we demonstrated that the custom-made 3D bioprinting systems can successfully fabricate core-shell structures (Fig. 2). The permeability, integrity and continuity of the alginate shell were tested by perfusing flows, and feasibility of layer by layer accumulation without the core had also been verified, which is the key step for the success of this experiment. The resolution of the bioprinted core-shell fibers are about 870 μm and that of the cell fiber in core are arranged from 242.89 μm to 527.49 μm. Although at current stage, printing resolution of this platform has no advantages compared to other bioprinting techniques; this can be improved by using smaller nozzles. Furthermore, we consider the high cell viability and sufficient cell-cell interactions within the system are the main advantages. Cell-laden core-shell tumor fibers were then produced with high cell viabilities both immediately after printing and when cultured *in vitro* for 5 days (Figs 3, 4). Proliferation rate of cells in the fibers was significantly higher than that of mixed group, but slower than that of 2D group. HE staining showed that cells in fibers contacted each other, and grew well, but cells mixed into alginate were isolated and fragmented (Fig. 5). Newly produced collagen, the most important composition of ECM, was verified by Masson staining (Fig. 5), and production of ECM was a fundamental biological feature of viable tissues. RFP/GFP tracing demonstrated that tumor cells and stromal cells distributed and arranged in fibrous structures, and cells stretched, contacted, and fused into continuous fibers (Fig. 6). Cell biomarkers confirmed the intrinsic characteristics of the tumor cells and stromal cells printed in fibers, and further illustrated the functionalities, such as production of self-assembled ECM of the cells (Fig. 7). Cell fibers exhibited heterogeneous tumor tissue-like spatial structures, morphologies and partial but most essential functions. Although it was not fully mimicking the tissues *in vivo*, the coaxial printed cell fiber is a better tumor model than cells mixed into alginate (Mixed group).

In order to further verify that the sufficient interactions between tumor cells and stromal cells, RFP transcription and expression were detected by qRT-PCR and confocal microscopy (Fig. 8). Results showed that RFP transcription of the cell fibers was significantly higher than the control groups (2D group and mixed group). Presence of the RFP had been observed by laser scanning confocal microscope. Cell fusion demonstrated excellent tumor-stroma cell interactions in coaxial bioprinted cell fibers.

Coaxial bioprinted cell fibers provide appropriate support and microenvironment for cell survival and cell-cell interactions. It is an alternative *in vitro* model mimicking *in vivo* tumors for tumor research. However, there are several limitations of this study, such as (1) the strength of the shell are not completely controllable and hard to maintain over a long period of time, and cells may not have enough time to self-assemble fibers with sufficient strength; preventing Ca^2+^ from being extracted by phosphate anion (PO4^3−^) in the culturing medium may be a potential creative method to maintain strength which needs further investigation. (2) the spatial precision of the 3D bioprinted structures is a drawback, especially after decrosslinking the alginate shell; (3) before dissociation of the shell, apoptotic cell debris cannot be removed, and it may affect cell activity. Proper selection of the time point for dissociation will help to reduce the accumulation of apoptotic cell debris as early as possible.

## Conclusion

In conclusion, coaxial 3D biofabricated multicellular self-assembled heterogeneous tumor tissue-like fibers provided preferable 3D tumor models for studying tumor microenvironment *in vitro*, especially for tumor-stromal interactions.

## Methods and Materials

### Cell culture and lentiviral transduction

Glioma stem cell line, GSC23 was kindly provided by the MD Anderson Cancer Center, University of Texas^35^. HMSCs were bought from ScienCell Research Laboratories (Sciencell, Cat. #7500, CA, USA). Similar to previously reported methods^36^, GSC23 cells were transfected with red fluorescence protein (RFP) gene, and hMSCs were transfected with green fluorescence protein (GFP) gene. In another method, GSC23 cells were also transfected with a lentiviral vector GV348 (sequence element: Ubi-MCS-SV40-puromycin) containing LOXP-STOP-LOXP-RFP gene, and MSCs were transfected with a lentiviral vector GV348 containing a CRE enzyme gene. The RFP, GFP and CRE-LOXP lentiviral vectors were packaged by Shanghai Genechem Co., Ltd (Shanghai, China). MSCs were cultured in Mesenchymal Stem Cell Medium (MSCM, Sciencell, Cat. #7501). GSC23 cells were maintained in Dulbecco’s Modified Eagle Medium/F12 (DMEM/F12), containing 20 ng/ml basic fibroblast growth factor (bFGF), 20 ng/ml epidermal growth factor (EGF), B27 supplement (50X), 2 mmol/l L-glutamine (100X), MEM vitamin solution (100X) and 100 mM sodium pyruvate (100X) (all from Gibco, Carlsbad, CA, USA).

### Materials

Sodium alginate was purchased from WaKo (199–09961, 500–600 cP, WaKo Pure Chemical Industries, Ltd., Osaka, Japan). Alginate was dissolved into deionized water at the concentration of 3% (w/v) and placed in ultrasonic water bath at 37 °C for 12 h to get the sodium alginate sol solutions. Gelatin (5% w/v, Sigma-Aldrich) was added into alginate (Alginate/Gelatin, A/G) to improve the microenvironment of sol solutions for better cell survival^16^. The concentrations of sodium alginate and gelatin are optimally selected (Supplemental Fig. 2). Calcium chloride (CaCl_2_, Sigma-Aldrich, Shanghai, China) was dissolved into deionized water at a final concentration of 3% (w/v) (Supplemental Fig. 3). For de-crosslinking of alginate, 55 mM sodium citrate (Sigma-Aldrich) and 20 mM EDTA (Sigma-Aldrich) in 0.9% NaCl solution was prepared. Fibrinogen and thrombin were bought from Biosharp (Biosharp, Hefei, China). Blue (FD&C BLUE NO. 1) and red (FD&C red NO. 1) dye (Vidhi Dyestuff Manufacturing Limited, Tardeo, Mumbai, India) were added to the CaCl_2_ solution to distinguish the core and shell sections of the hollow filaments and identify their liquidity, structural integrity and permeability after perfusion.

### Coaxial bioprinting

Core-shell structures were bioprinted with a custom-made computer-controlled coaxial extruding bioprinting system. The print head contains two nozzles arranged in concentric circles and connected to different pumps, one to A/G hydrogel, and the other to high density cell suspension (1 × 10^7^/ml) in 3% fibrinogen (w/v) (CoF group). The extrusion speed was 15–30 ml/h for shell materials and 3–6 ml/h for the cell suspension core. The receiving platform was a tank containing 3% CaCl_2_ solution. After printing, core-shell structures were washed with 0.9% NaCl solution to remove excessive calcium ions and then placed in fresh medium. The same procedure was repeated for both the 2D and mixed control groups.

### Cell viability and proliferation

Cell viability was assessed by fluorescence live/dead viability assay kit (KeyGEN BioTECH, Nanjing, China) following the manufacturer’s instruction. Briefly, 8 μM propidium iodide (PI) and 2 μM Calcein-AM were mixed with phosphate-buffered saline (PBS). Cell fibers were incubated in the staining solution for 15 min in dark at room temperature. Live cells were stained green on the membrane and dead cells stained red on the nucleus. Images were acquired from fluorescence microscope. Living/dead cells were counted in 5 random sights of each sample at 200 × magnification (n = 3). Cell proliferation was evaluated via the Cell Count Kit-8 (CCK-8, Dojindo, Japan). Cell-laden fibers of identical length (50 mm) were washed and individual fibers were incubated in each well of 24-well plates with CCK-8 working solution (CCK-8: Fresh medium = 1:9) at 37 °C for 2 h. The solutions were transferred to a 96-well plate to read the optical density (OD) at wavelength of 450 nm (BioTek ELX800, VT, USA). This procedure was repeated every other day for 5 days and performed on the control groups as well. The OD of all three groups were normalized to day 1 and used for plotting and statistics^37^.

### Alginate shell removal

Over time, GSC23 cells and MSCs interacted with each other and self-assembled into heterogeneous tumor fibers. After 3 weeks of culturing, to remove the shell, tumor fibers were soaked into 55 mM sodium citrate and 20 mM EDTA (Sigma Aldrich, Shanghai, China) solution for 5 min according to a previously reported method^38^.

### Histological and immunohistological analyses

Bioprinted tumor fibers were fixed in 4% paraformaldehyde and paraffin-embedded. 3 μm thick sections were produced via a microtome (SLEE, Germany), and stained with H&E according to routine histology protocols. Masson staining (Solarbio, Beijing, China) was performed to identify the ECM (collagen fiber). Phalloidin was stained according to the instructions. To identify the biomarkers, immunofluorescence analysis was performed as described previously^39^. Primary antibodies were Nestin (ab22035), CD44 (ab157107), vimentin (ab92547), N-Cadherin (ab18203) (all from Abcam), and secondary antibodies were Alexa Fluor 488 and Cy3-labeled goat anti-rabbit and mouse IgG (Abbkine, California, USA). Cell nucleus were stained with 4′,6-diamidino-2-phenylindole (DAPI, Solarbio, China). Expression of cell surface markers was observed with a fluorescence microscope (Olympus IX51; Tokyo, Japan), and images were captured and merged using software provided with the microscope. GBM is a paraffin specimen of glioblastoma originating from our laboratory tissue bank, and the xenograft tumor is a paraffin-embedded tissue derived from glioma stem cells transplanted *in situ* in balb/c nude mice, and they were used as controls. The patient materials (GBM specimen) was obtained from an informed patient. All experimental methods and protocols were carried out in accordance with the approved guidelines by the Research Ethics Committee of the Second Affiliated Hospital of Soochow University (Suzhou, China). Animal xenograft tumor was established in our lab, and all animal experiments were performed in strict accordance with the principles and procedures of the Animal Care Committee of the Second Affiliated Hospital of Soochow University.

### Fluorescence observation

To evaluate the interactions between tumor cells and stroma cells in the fibers, RFP/GFP tumor/stroma cell fibers were observed directly under the fluorescence microscope. GSC23-LOXP cells and MSC-CRE cells were observed under confocal laser scanning microscope (CLSM) to detect the presence of RFP-expressing cells. Tumor fiber were fixed and stained with DAPI (Solarbio) and phalloidin (AAT Bioquest, Inc). As controls, the same procedure was also performed on 2D and mixed control samples.

### Quantitative real-time PCR (qRT-PCR)

For qRT-PCR, tumor fibers were dissolved in dissociation solution as described above at day 7 and cell fibers were centrifuged and resuspended in Trizol (Invitrogen, 15596–026). Total RNA was isolated and transcribed into cDNA according to the protocol. Primer sequences for WPRE gene in the viral vector of CRE and LOXP-STOP-LOXP-RFP is F: 5′ AATTCCGTGGTGTTGTCG 3′, R: 5′ AAGGTCCGCTGGATTGAG 3′; GAPDH F: 5′ GGCCTCCAAGGAGTAAGAAA 3′, R: 5′ GCCCCTCCTGTTATTATGG 3′; RFP F: 5′ CCCGTAATGCAGAAGAAGAC 3′, R: 5′ GCTTGGAGTCCACGTAGTAG 3′. Following this step, PCR amplification was performed, with the condition of 50 °C 2 min; 95 °C 15 s; 60 °C 32 s; reading plate; 40cycles. Each sample was repeated 3 times.

### Statistical analysis

Each experiment was repeated 3 times and results are presented as mean ± SD. Comparison between two groups was performed using the Student’s t-test. Statistical significance was attained at P < 0.05. The results were analyzed and exported using GraphPad Prism 5.0 software.

## Electronic supplementary material

Supplemental materials
