# Supplementary material for: Coaxial 3D bioprinting of self-assembled multicellular heterogeneous tumor fibers
Source: Sci Rep. 2017 May 3;7:1457. doi: 10.1038/s41598-017-01581-y (PMC5431218; doi:10.1038/s41598-017-01581-y)
Supplement: Supplementary file 1 — Supplemental materials [file 41598_2017_1581_MOESM1_ESM.pdf]

Coaxial 3D bioprinting of self-assembled multicellular heterogeneous tumor fibers

Xingliang Dai[1], Libiao Liu[2], Jia Ouyang[1], Xinda Li[2], Xinzhi Zhang[3], Qing Lan[1*], Tao Xu[2, 4*]

1. Department of Neurosurgery, the Second Affiliated Hospital of Soochow University, Suzhou, 215004, China
2. Department of Mechanical Engineering, Biomanufacturing Center, Tsinghua University, Beijing, 100084, China
3. Medprin Biotech GmbH, Gutleutstraße 163-167, Frankfurt am Main, 60327, Germany
4. Department of Precision Medicine and Healthcare, Tsinghua-Berkeley Shenzhen Institute, Shenzhen 518055, China

* Corresponding author: Tao Xu,
Email: taoxu@mail.tsinghua.edu.cn
Qing Lan,
Email: szlq006@163.com

Supplemental Figure 1

As shown on Fig.8D, RFP was observed in cytoplasm of the cells in CoF group, while it was almost invisible in the control group (mixed group), as shown below.

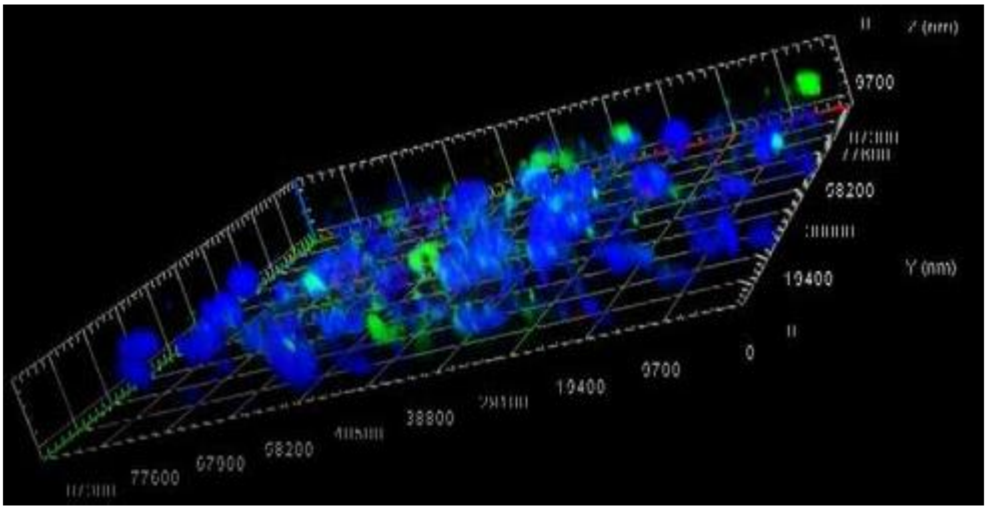

Supplemental Figure 1: Confocal image of the control group, RFP would be in red if there was any. Cytoskeletal structures were stained green by phalloidin and cell nucleus were stained blue by DAPI.

Supplemental Figure 2

At room temperature (25°C), the viscosity of the A/G shell (3 wt% alginate mixed with 5 wt% gelatin) was (82.00±7.00) mPa.s. Greater than this concentration the system would become almost solid-like, and less than this value the alginate would not be able to ensure the integrity of the shell.

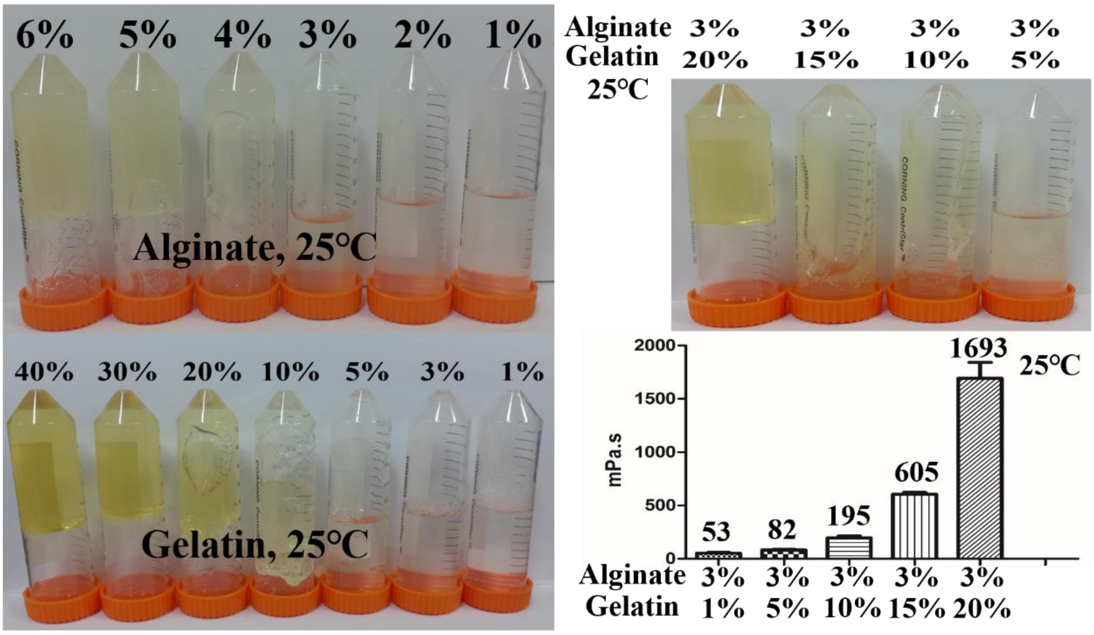

Supplemental Figure 2: Viscosity of the A/G shell. At room temperature (25°C), 3 wt% alginate was mixed with gelatin of different concentrations; their viscosity was (53.00±5.57) mPa.s, (82.00±7.00) mPa.s, (195.00±19.97) mPa.s, (605.00±17.35) mPa.s and (1693.00±149.94) mPa.s, respectively.

Supplemental Figure 3

We tried to study the effect of $CaCl_2$ concentration on cell viability. We use fibroblast L929 incubated with $CaCl_2$-DMEM (10% FBS) solution for 24 hours. When $CaCl_2$ concentration was greater than 0.3% the impact on cell viability was significant. However, the crosslinking speed and time of alginate gelation with 0.3% $CaCl_2$ cannot meet the extrusion requirements. Thus, based on previous studies and our own experience, a slightly higher concentration of $CaCl_2$ (3%) [1, 2] was used to speed up crosslinking and decrease the crosslinking time.

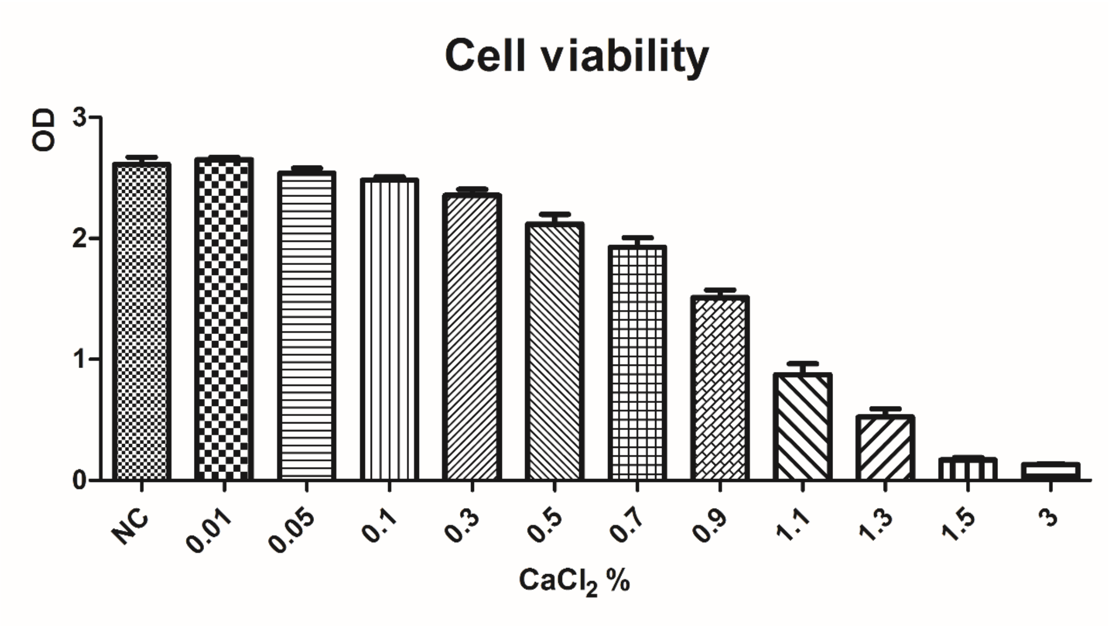

Supplemental Figure 3: Cell viability of L929 cells incubated with different concentrations of $CaCl_2$ for 24h. 0.01%, 0.05%, 0.1%, 0.3%, 0.5%, 0.7%, 0.9%, 1.1%, 1.3%, 1.5% and 3% $CaCl_2$, compared to Blank group (NC), relative cell viability was 101.45%, 97.17%, 94.93%, 90.23%, 81.11%, 73.77%, 57.81%, 33.44%, 20.07% and 6.45%.

[1] Zhao Y, et al. Three-dimensional printing of Hela cells for cervical tumor model in vitro. *Biofabrication* 6:035001 (2014).

[2] Dai X, Ma C, Lan Q, Xu T. 3D bioprinted glioma stem cells for brain tumor model and applications of drug susceptibility. *Biofabrication* 8:045005 (2016).
